# Dual-Force ISOMAP: A New Relevance Feedback Method for Medical Image Retrieval

**DOI:** 10.1371/journal.pone.0084096

**Published:** 2013-12-31

**Authors:** Hualei Shen, Dacheng Tao, Dianfu Ma

**Affiliations:** 1 State Key Laboratory of Software Development Environment, School of Computer Science and Engineering, Beihang University, Beijing, China; 2 Center for Quantum Computation and Intelligent Systems, Faculty of Engineering and Information Technology, University of Technology, Sydney, NSW, Australia; 3 School of Computer and Information Engineering, Henan Normal University, Xinxiang, China; Institute of Psychology, Chinese Academy of Sciences, China

## Abstract

With great potential for assisting radiological image interpretation and decision making, content-based image retrieval in the medical domain has become a hot topic in recent years. Many methods to enhance the performance of content-based medical image retrieval have been proposed, among which the relevance feedback (RF) scheme is one of the most promising. Given user feedback information, RF algorithms interactively learn a user’s preferences to bridge the “semantic gap” between low-level computerized visual features and high-level human semantic perception and thus improve retrieval performance. However, most existing RF algorithms perform in the original high-dimensional feature space and ignore the manifold structure of the low-level visual features of images. In this paper, we propose a new method, termed dual-force ISOMAP (DFISOMAP), for content-based medical image retrieval. Under the assumption that medical images lie on a low-dimensional manifold embedded in a high-dimensional ambient space, DFISOMAP operates in the following three stages. First, the geometric structure of positive examples in the learned low-dimensional embedding is preserved according to the isometric feature mapping (ISOMAP) criterion. To precisely model the geometric structure, a reconstruction error constraint is also added. Second, the average distance between positive and negative examples is maximized to separate them; this margin maximization acts as a force that pushes negative examples far away from positive examples. Finally, the similarity propagation technique is utilized to provide negative examples with another force that will pull them back into the negative sample set. We evaluate the proposed method on a subset of the IRMA medical image dataset with a RF-based medical image retrieval framework. Experimental results show that DFISOMAP outperforms popular approaches for content-based medical image retrieval in terms of accuracy and stability.

## Introduction

Medical image interpretation is a process which incorporates subjective perception and objective reasoning. Typically, radiologists obtain superficial visual features from medical images and render diagnostic conclusions based on personal knowledge and experience. Due to differences of perception, training and fatigue, different conclusions about the same medical image will be drawn by different professionals or by the same professional under different circumstances [1], [2]. The goal of content-based medical image retrieval (CBMIR) is to enable radiologists to make better diagnosis about a given case by retrieving similar cases from a variety of semantically annotated medical image archives.

**Figure 1:**
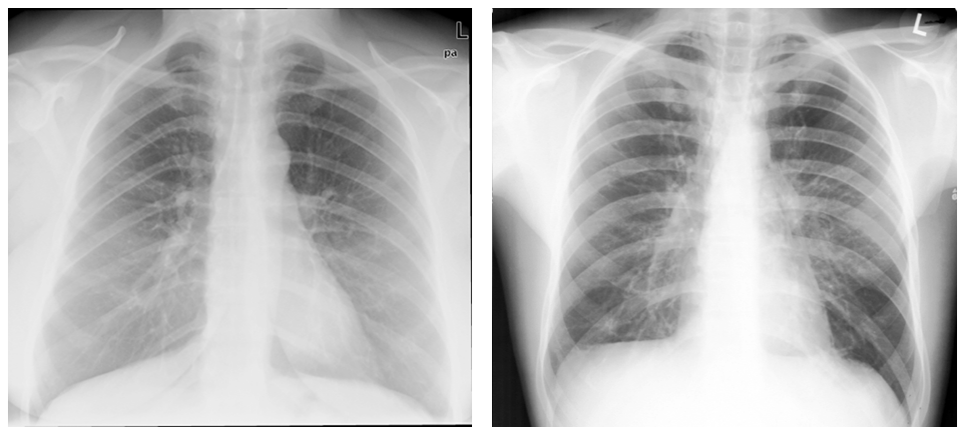
Visually similar medical images contain different semantic meanings. The chest radiographs shown in this figure have a similar visual appearance but belong to different semantic categories. Images are taken from IRMA medical image dataset.

**Figure 2:**
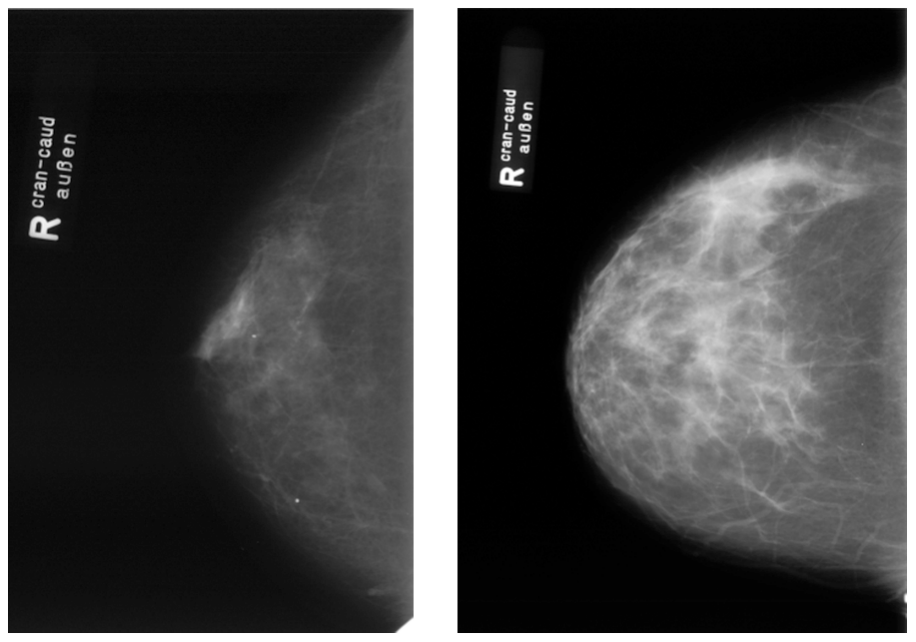
Semantically-alike medical images have a different visual appearance. The mammograms shown in this figure belong to the same semantic category, though they have a different visual appearance. Images are taken from IRMA medical image dataset.

It is well-known that “semantic gap” is one of the issues faced by content-based image retrieval (CBIR). The fact that medical images contain varied, rich and subtle visual features [3] is an additional challenge to the use of CBIR in radiology. Unlike from regular image understanding, medical image diagnosis is dependent on case-specific interpretation. It is common for visually similar medical images to convey different semantic meanings, while semantically-alike images have different visual features. Let us take medical images obtained from IRMA medical image dataset [4] as an example. The IRMA medical image dataset is a widely used test bed for performance evaluation of CBMIR [5]–[8]. The new version of IRMA dataset [4] contains 12,677 fully annotated gray value radiographs in a training set. These images are categorized into 193 classes according to a mono-hierarchical multi-axial classification standard called the IRMA coding system [9]. The system classifies a medical image from four orthogonal axes: imaging modality, body orientation, body region examined and biological system examined. **Figure 1** and **Figure 2** illustrate the scenario of semantic gap. As shown in **Figure 1**, two chest radiographs have a similar visual appearance, but their semantic meanings are different. The IRMA code [9] of the left image is “1123-127-500-000”, while the IRMA code of the right image is “1123-110-500-003”. By contrast, though their visual appearance is different, the two mammograms shown in **Figure 2** have the same IRMA code “1124-310-610-625”.

Relevance feedback (RF) is a promising solution to fill the semantic gap in CBIR [10]. Under the assumption that every user’s need is different and time varying [11], [12], RF provides a *user-in-the-loop* mechanism to allow a user to interact with the retrieval system to refine the retrieval results. The basic process of RF in CBIR is as follows: 1) the retrieval system returns the initial retrieval results to the user; 2) the user labels query-relevant images and query-irrelevant images as positive feedback and negative feedback, respectively; 3) based on the labeled feedback, the retrieval system learns to improve the retrieval performance and returns new results; 4) if the user is satisfied with the new results, the RF process ends; otherwise, go to 2).

Over the past decades, many representative RF approaches have been proposed in the context of CBIR [13]–[23]. A comprehensive survey of these methods can be found in [11], [24]. In [25], RF methods are categorized into four groups: subspace selection-based schemes, support vector machine (SVM)-based schemes, random sampling-based schemes and feature reweighting-based schemes. Performance evaluations of several RF approaches are reported in [26], [27].

Many RF methods have also appeared in CBMIR in recent years. Rahman et al. [28] utilized positive feedback to update the optimal query point for medical image retrieval. They proposed a RF-based dynamic similarity fusion approach for biomedical image retrieval [29] in which RF information is utilized to reweight features at each iteration. Xu et al. [30], [31] utilized RF to update feature weights for X-ray image retrieval. To solve the small sample size problem, Hoi et al. [32] proposed a method called semi-supervised SVM batch mode active learning for both medical and regular image retrieval. In addition, Ko et al. [33] integrated the RF scheme into CBMIR to boost retrieval performance. Though the approaches mentioned above achieve promising results, there is room for performance enhancement because most of these methods do not consider the manifold structure of low-level image features.

In this paper, we formulate a new RF method termed dual-force ISOMAP (DFISOMAP) for CBMIR. DFISOMAP is proposed in the context of precisely exploring the manifold structure of low-level image visual features [34]–[36]. DFISOMAP operates in the following three stages: 1) the local geometry preservation stage, 2) the margin maximization stage, and 3) the similarity propagation stage. First, the local geometry of the positive examples in the high-dimensional feature space is preserved according to the isometric feature mapping (ISOMAP) criterion [37]. To precisely model the geometric structure of positive examples in the low-dimensional embedding, a reconstruction error constraint according to locally linear embedding (LLE) [38] is also added. Second, negative examples are pushed away from positive examples by a force driven by the maximization of average pairwise distances between the positive and negative examples. Finally, negative examples are pulled into the negative sample set by another force generated by similarity propagation. We conduct experiments to demonstrate the effectiveness of DFISOMAP. Compared to conventional RF methods, e.g., linear discriminant analysis (LDA) [39], locality preserving projections (LPP) [40], biased discriminant analysis (BDA) [21], constrained similarity measure using support vector machine (CSVM) [18], ISOMAP and exponential locality preserving projections (ELPP) [41], DFISOMAP differ in the following ways: 1) DFISOMAP precisely preserves the geometric structure of positive feedback examples, and 2) DFISOMAP does not suffer from the undersampling problem.

## Dual-Force ISOMAP

In this section, we detail the proposed DFISOMAP. To better present the method, **Table 1** lists important notations used in this paper.

**Table 1:** Important notations used in this paper.

Notation	Description	Notation	Description
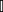	medical image dataset	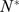	similarity matrix
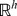	high-dimensional ambient space	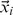	the 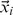 medical image contained in 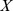
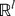	low-dimensional embedding	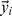	the 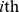 medical image contained in 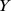
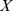	relevance feedback set in 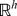	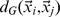	geodesic distance between 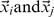
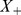	positive relevance feedback set	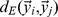	Euclidean distance between 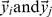
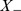	negative relevance feedback set	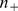	size of 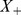
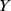	relevance feedback set in 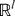	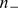	size of 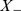
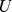	projection matrix, 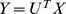	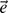	identity vector
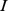	identity matrix	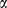	trade-off parameter
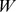	reconstruction coefficient matrix in LLE	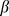	trade-off parameter
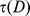	linear product matrix of 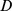	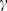	margin factor

Consider a set of medical images 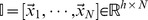 in low-level feature space, and a query image 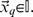 Following the query-by-example paradigm of the CBIR system, there are top 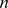 returned images for each query, from which we obtain 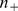 images which are from the same semantic class as 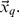 We term them *positive examples*: 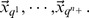 Putting these examples together, we get a *positive feedback set* 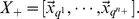 Meanwhile, we obtain 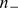 images, which are from different semantic classes with respect to 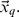 We term them *negative examples*: 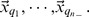 Putting these examples together, we get a negative feedback set 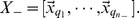 The *relevance feedback set* 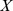 is constructed by putting 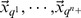 and 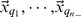 together as 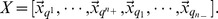 where the first 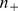 are positive examples and the remaining 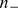 are negative examples, 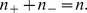 For convenience, we use 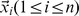 to represent all examples, and denote 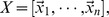 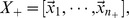 and 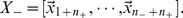


DFISOMAP assumes that medical images lie on a low-dimensional manifold 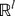 and are artificially embedded in a high-dimensional ambient space, i.e., the low-level feature space 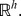 The objective of DFISOMAP is to learn a mapping function 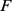 from 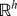 to 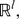 based on the relevance feedback set 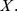 The learned mapping 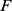 should effectively separate positive examples from negative examples. For simplicity, we assume that 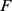 is linear. The problem of DFISOMAP is then converted to find a projection matrix 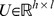 that maps 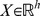 to 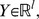 i.e., 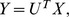 where 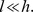 Here, each column of 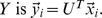


DFISOMAP operates in three stages which containing two forces to separate negative examples from positive examples. In the first stage, the local geometric structure of the positive examples is preserved according to the ISOMAP criterion [37]. To make the local geometry preservation more precise, an error reconstruction constraint is added. This stage is termed “*local geometry preservation*”. In the second stage, a margin maximization function is defined to maximize the gap between positive examples and negative examples. The margin maximization function acts as a force to push negative examples away from positive examples, and this stage is termed “*margin maximization*”. In the final stage, termed “*similarity propagation*”, the similarity propagation technique [42] is employed to build a similarity matrix which quantifies similarities between the intraclass examples contained in the relevance feedback set. Based on the similarity matrix, the distance between the intraclass examples is minimized to shrink the distance between image pairs from the same semantic class. The procedure acts as another force to pull negative examples away from positive examples.

### 2.1. Local Geometry Preservation

ISOMAP preserves the local geometry of positive examples by the following objective function [37]



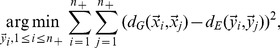
(1)


where 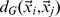 is the geodesic distance between image 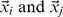 in high-dimensional space 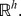 And 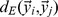 is the corresponding Euclidean distance between image 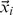 and 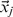 in low-dimensional embedding 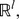 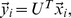 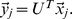


Let us denote 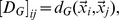 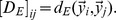 Where 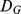 and 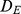 are 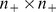 matrices. According to [37], 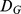 and 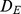 can be converted to inner product matrix 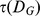 and 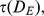 respectively. Operator 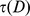 is defined as



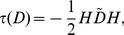
(2)




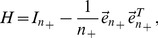
(3)




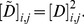
(4)


where 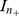 is an 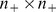 identity matrix, 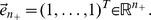 Thus, equation (1) can be transformed to



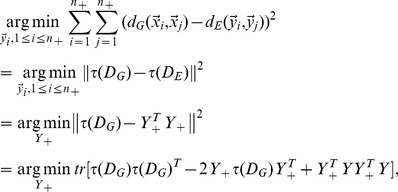
(5)


where 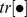 stands for the trace operator, 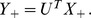 Assuming that 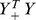 is a constant matrix, equation (5) can be converted to



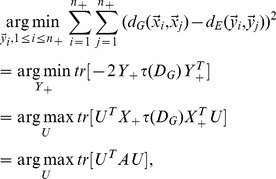
(6)


where 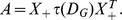


To minimize reconstruction error of the local geometry preservation presented above, we further assume each 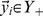 can be reconstructed by its neighbors. Thus, we have



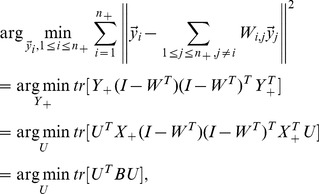
(7)


where 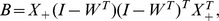 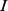 is an 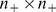 identity matrix. 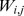 is obtained via locally linear embedding (LLE) [38]:



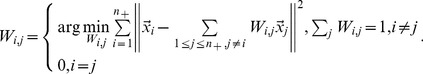
(8)


Putting equation (6) and (7) together, we obtain the objective function for local geometry preservation



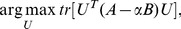
(9)


where 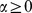 is the trade-off parameter.

### 2.2. Margin Maximization

In the low-dimensional embedding, we expect that the average pairwise distances between negative and positive feedback examples will be as large as possible, and the average pairwise distances among positive feedback examples will be as small as possible, i.e.,



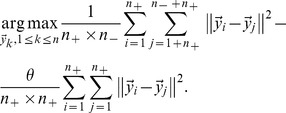
(10)


Where 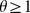 is the gap factor. Considering 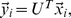 equation (10) reduces to:



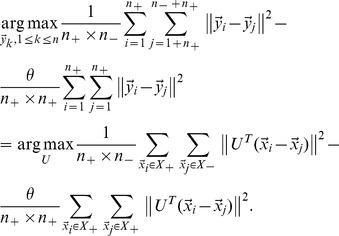
(11)


### 2.3. Similarity Propagation

Equation (11) only takes into account the distances between the positive examples and coarsely treats negative examples. To remedy this, we need the average pairwise distance among the intraclass examples to be rendered as small as possible.

The straightforward way to shrink the pairwise distance between interclass examples is to minimize the average weighted square distance between all sample pairs 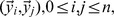 in the low-dimensional embedding:



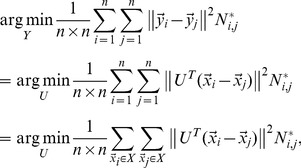
(12)


where 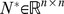
is termed similarity matrix.

In this paper, we define
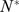
as



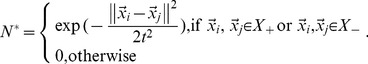
(13)



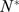
quantifies the similarity relationship among positive and negative examples, respectively. In our implementation, we set
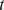
as 0.5.

Putting equation (11) and (12) together, we have



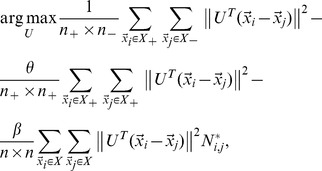
(14)


where 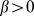 is the trade-off parameter. Let us denote



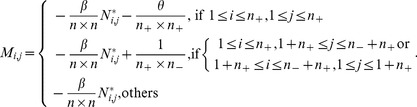
(15)


Then equation (14) can be rewritten as



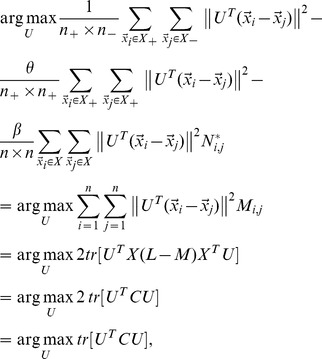
(16)


where 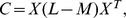 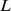
is a diagonal matrix,
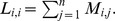


### 2.4. Objective Function

Combining equation (9) with (16), we obtain the objective function of DFISOMAP



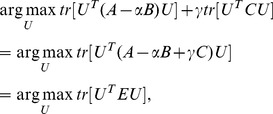
(17)


where 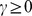 is the margin factor, 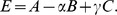


Because the real matrix 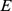 is symmetric (the proof is given in Appendix S1), 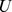 can be solved by standard eigenvalue decomposition on 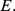 By imposing 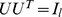 on (17), 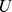 is formed by the 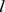 eigenvectors associated with the first 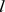 largest eigenvalues.

## CBMIR Framework

**Figure 3:**
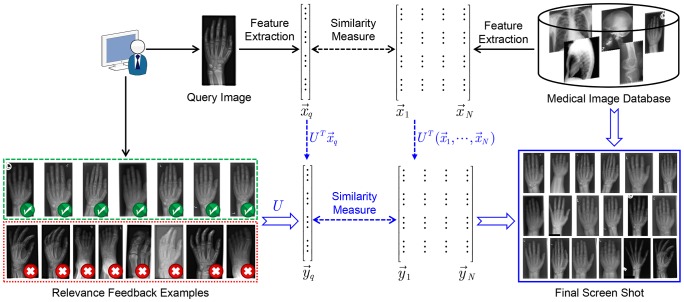
Relevance feedback-based medical image retrieval framework.

We utilize the framework depicted in **Figure 3** for CBMIR. Any RF feedback algorithm can be integrated into this framework. As shown in this figure, when a query image is provided, its low-level visual features are extracted. All images contained in the medical image database are then sorted in ascending order according to their distance from the query image measured by Euclidean metric. If the user is not satisfied with the result, s/he labels some semantically relevant images as positive feedback examples and some semantically irrelevant images as negative feedback examples. Based on these feedback examples, a RF model can be trained. All images, including the positive feedback, the negative feedback and the remaining images contained in the medical image database, are re-sorted based on the updated similarity metric and the top-ranked images are returned. If the user is not satisfied with the result, the RF process is repeated.

For DFISOMAP, we learn a projection matrix 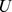 according to equation(17). Then we use 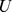 to project all the images to the low-dimensional embedding. In the projected embedding, each image is re-sorted in ascending order with respect to its Euclidean distance from the query image and the top-ranked images are returned to the user. The RF procedure stops when the user is satisfied with the results.

We use LBP [43], SIFT [44] and pixel intensity descriptors respectively to extract features from the medical image. For the LBP descriptor, we divide each medical image into 3×3 equal regions. On each region, a 59-bin LBP histogram is built. Then we concatenate these 59-bin LBP histograms into a 531-D vector. For the SIFT and intensity descriptors, we follow bag of features [45] scheme to represent the image. In detail, we first densely sample each image with SIFT and the intensity descriptor, respectively. We set the sampling space as 8, and the patch size as 16×16. Then we use K-means clustering to learn two 500-word dictionaries, i.e., SIFT and intensity visual word dictionary. Finally, for each image, we obtain a 500-bin SIFT and intensity histogram, respectively.

We represent each image by concatenating the 531-bin LBP histogram, 500-bin SIFT histogram and 500-bin pixel intensity histogram into a 1531-D long vector. To get rid of redundant information contained in the concatenated vector and reduce the computational complexity in the next section, we normalize the concatenated 1531-D vector into a normal distribution with zero mean and one standard deviation. Then we use principal component analysis (PCA) to reduce the normalized vector to a 500-D feature vector.

## Performance Evaluation

In this section, we report performance of the proposed DFISOMAP for CBMIR comparing with that of other methods, i.e., LDA, LPP, BDA, CSVM, ISOMAP, LLE and ELPP.

This section is organized as follows. In section 4.1, we introduce the dataset used for evaluation. Section 4.2 presents experimental setup. In section 4.3, we compare DFISOMAP with other RF approaches using mean average precision (MAP) and standard deviation (SD). Section 4.4 reports performance evaluation results of RF methods in terms of precision and recall. Finally, we explore effects of parameters on the performance of DFISOMAP in section 4.5.

### 4.1. IRMA Medical Image Dataset

**Figure 4:**
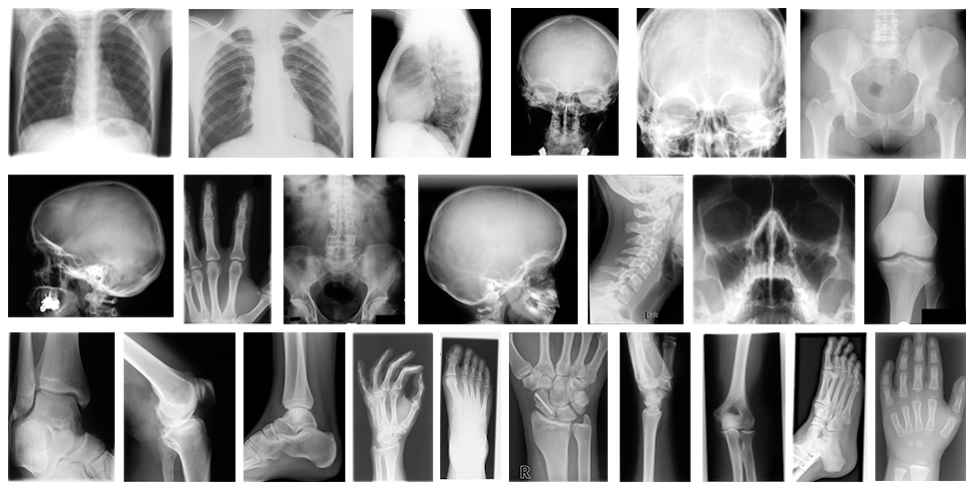
Examples of images in IRMA medical image testbed.

**Figure 5:**
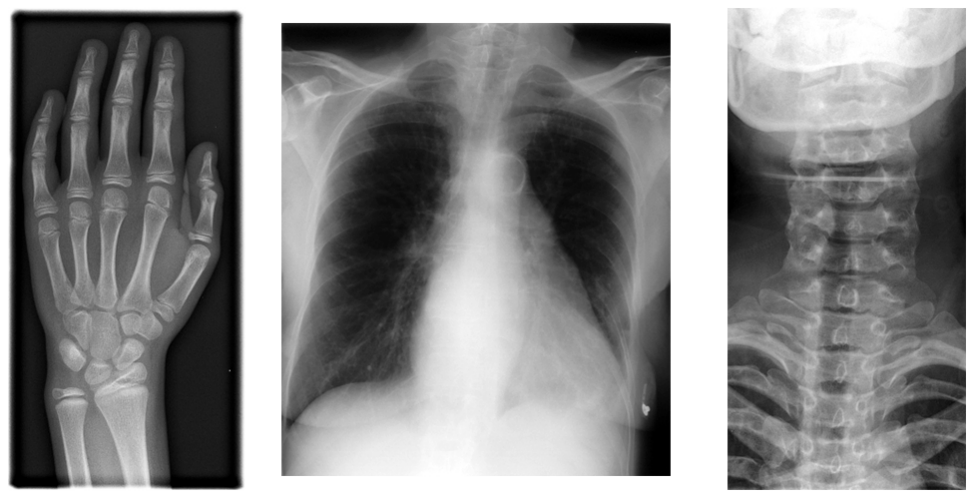
Examples of query image.

The IRMA medical image dataset is widely used for CBMIR evaluation. In our experiment, we select the first 57 categories from the new version of IRMA dataset as test bed. The selected images contain a total of 10,902 images. **Figure 4** shows example images from the dataset. **Figure 5** illustrates three query images.

### 4.2. Experimental Setting

We conduct 338 independent experiments to evaluate performance of DFISOMAP and other RF methods. In detail, we randomly select 338 images from the IRMA data set as query examples. These images belong to different IRMA categories. In general, five or six images are selected from each IRMA category. In initial retrieval, for each query sample, there are five to eight relevant images in top30 ranked results. For each selected image, a “leave one out” query is conducted: Rest images contained in the data set are ranked according to their Euclidean distance to the query sample.

Different RF algorithms are embedded into the framework depicted in **Figure 3**. The RF process is automatically performed by the computer. A computer-simulated query for each query image is performed on all the other 10,901 images contained in the dataset. The computer marks all query relevant images as positive feedback in the top 30 images and the rest as negative feedback. In general, we have between two and eight images as positive feedback. The procedure is close to a real-world application scenario, because typically the user does not want to label many feedback examples in the iteration process. We set the number of RF iterations as 10. For the first iteration, the returned images are ranked according to their Euclidean distance from the query image. Starting from the second iteration, different RF algorithms learn different projection matrices 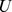 based on positive and negative feedback, respectively. In the projected low-dimensional embedding, other images in the dataset are re-ranked according to their Euclidean distance from the query image.

We parameterize the settings of all baseline methods according to the descriptions in corresponding papers. In the experiments, the parameters of different methods are tuned to obtain the best results. For CSVM, we choose the Gaussian kernel 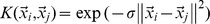 with 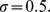 LibSVM [9] is utilized to achieve an optimal hyperplane to separate negative and positive examples. For ELPP, we set parameters as what is described in [41].

### 4.3. Performance Evaluation Using MAP and SD

In this section, we use MAP and SD to measure the performance of DFISOMAP and other RF algorithms. MAP is the mean of average precision values of the 338 independent queries. MAP value measures the retrieval precision of RF algorithms. SD value is computed from AP values of the 338 independent queries. SD value assesses the stability of RF algorithms.

**Figure 6:**
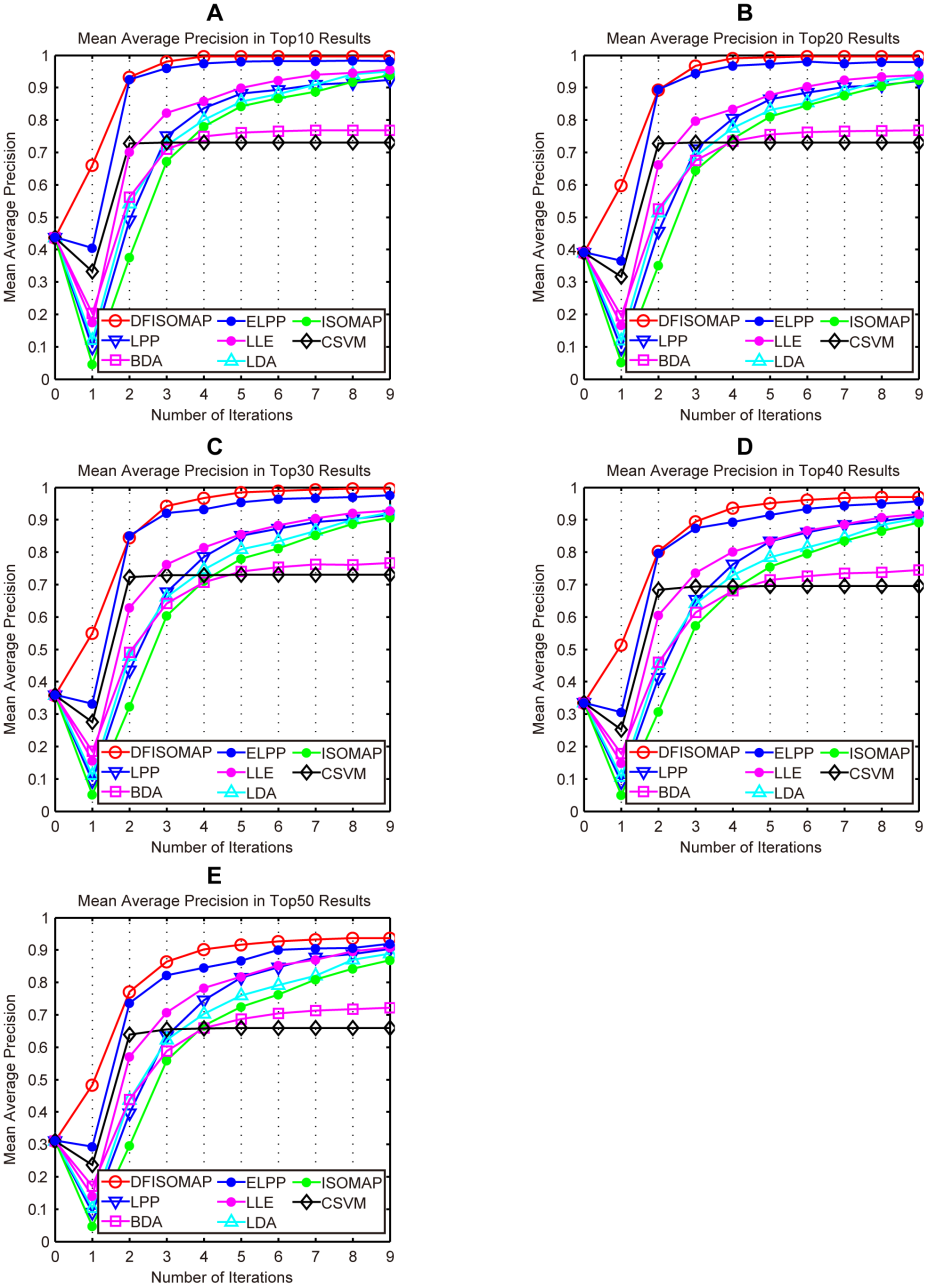
MAP values of DFISOMAP, LPP, BDA, ELPP, LLE, LDA, ISOMAP and CSVM. Subfigures (A), (B), (C), (D) and (E) detail MAP values in the top 10, top 20, top 30, top 40 and top 50 results, respectively.

**Figure 7:**
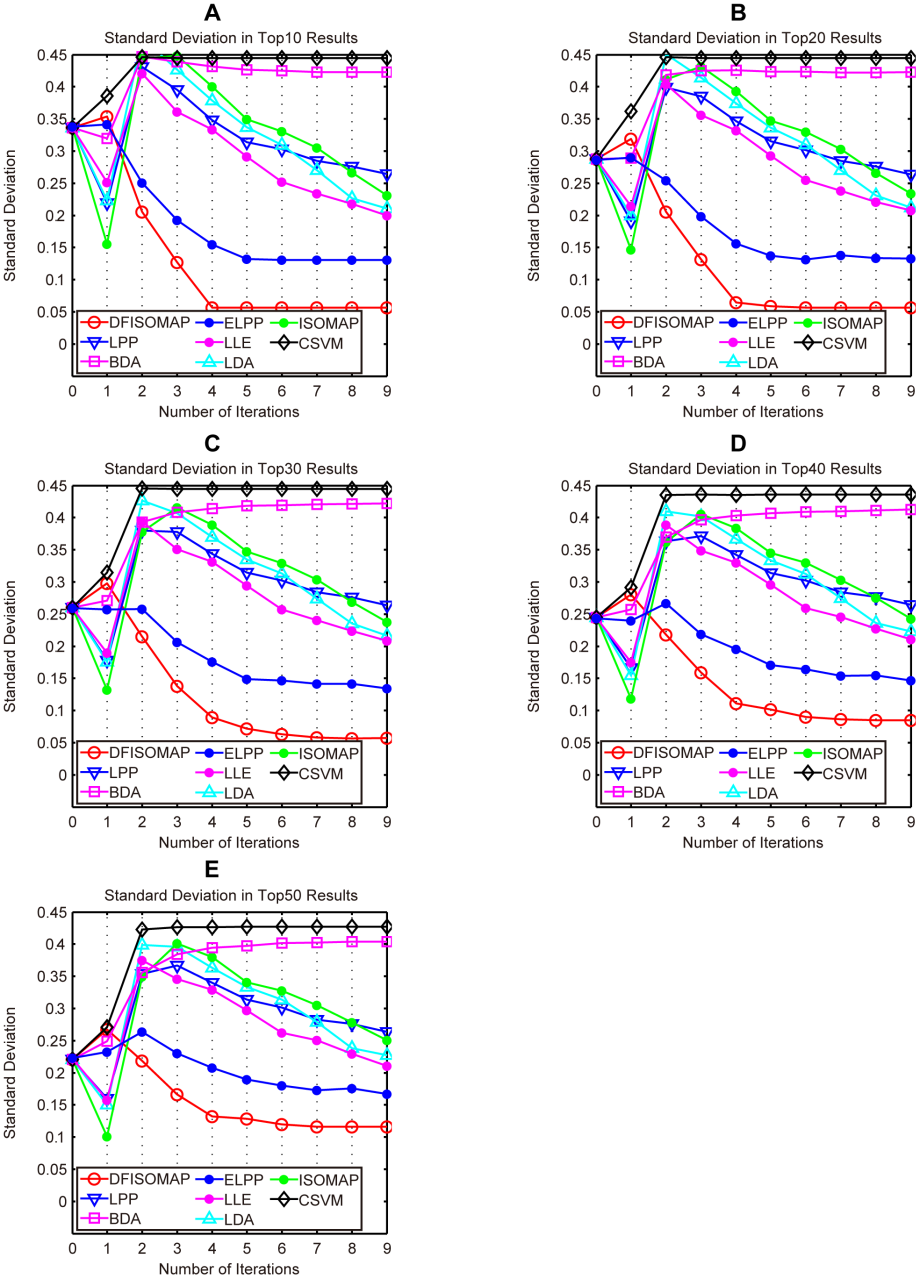
SD values of DFISOMAP, LPP, BDA, ELPP, LLE, LDA, ISOMAP and CSVM. Subfigures (A), (B), (C), (D) and (E) detail SD values in the top 10, top 20, top 30, top 40 and top 50 results, respectively.

**Figure 6** and **Figure 7** illustrate performance of the proposed DFISOMAP compared to LDA, LPP, BDA, CSVM, ISOMAP, LLE and ELPP-based RF algorithms. In **Figure 6**, subfigures (A), (B), (C), (D) and (E) show MAP values for the top 10, 20, 30, 40 and 50 results, respectively. The eight curves in each of these subfigures illustrate performance of the RF algorithms. The x-coordinate represents number of iterations, which varies from 0 to 9. Iteration 0 represents the initial retrieval measured by Euclidean distance in the high-dimensional feature space without RF, while iteration 1 refers to the first round RF based on feedback examples labeled in the 0th iteration, and similarly other iterations (from iteration 2 to 9). The y-coordinate indicates MAP values of different RF algorithms after each iteration. In **Figure 7**, subfigures (A), (B), (C), (D) and (E) detail SD values in the top 10, 20, 30, 40 and 50 results, respectively. SD indicates stability of the RF algorithm: the smaller the SD value, the more stable the algorithm.

From the figure we can see that, in all experiments, and after any number of iterations, the proposed DFISOMAP consistently outperforms other conventional RF algorithms in terms of MAP. The DFISOMAP also shows good stability, as demonstrated by the SD value and tendency of the SD curve. At each level (top 10 to 50), it can be seen that SD values of DFISOMAP for further iterations decrease after one iteration, and are much lower than those of other RF algorithms.

### 4.4. Performance Evaluation Using Precision and Recall

In this section, we utilize average precision (AP) and average recall (AR) to evaluate performance of DFISOMAP and other methods. In the context of CBMIR, precision refers to percentage of relevant medical images in top retrieved results. AP is calculated as the averaged precision values obtained via all queries. And recall refers to percentage of relevant medical images in all relevant examples contained in the test bed. AR is averaged recall values of all queries.

**Figure 8:**
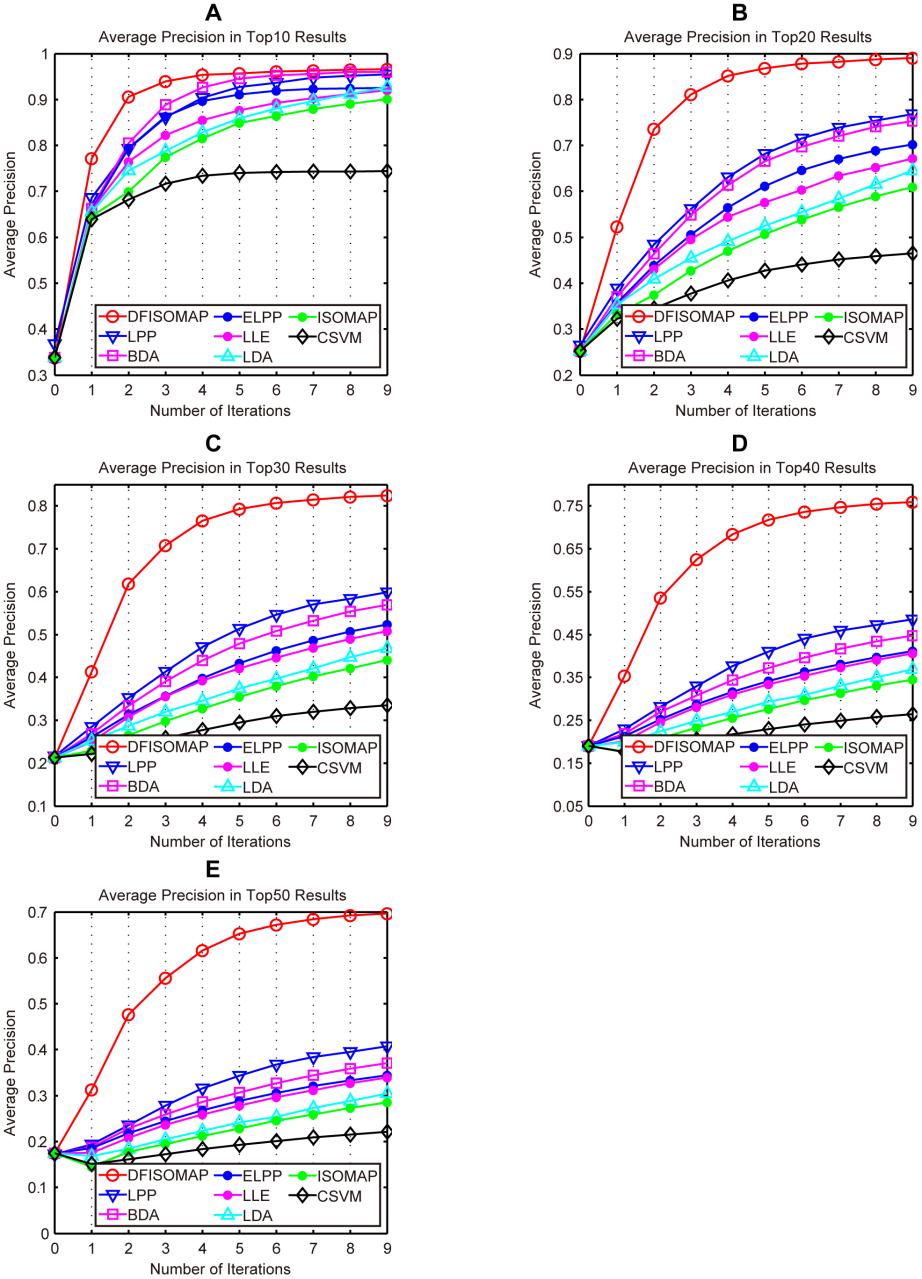
AP of DFISOMAP, LPP, BDA, ELPP, LLE, LDA, ISOMAP and CSVM. Subfigures (A), (B), (C), (D) and (E) detail AP in the top 10, 20, 30, 40, and 50 results, respectively.

**Figure 8**, **Table 2** and **Table 3** show AP of different methods. In detail, **Figure 8** (A), (B), (C), (D) and (E) present AP of different methods in the top 10, 20, 30, 40, and 50 results, respectively. As we can see from the figure, it is evident that DFISOMAP subsequently outperforms other algorithms. Details of the AP values of top ranked results for different approaches after the fifth and ninth feedback are presented in **Table 2** and **Table 3**, respectively. From these two tables, we can draw the conclusion that DFISOMAP achieves more promising results compared with other methods.

**Table 2:** Average precision of top ranked results for different methods after fifth feedback.

Methods	top10	top20	top30	top40	top50
**DFISOMAP**	**0.9571**	**0.8676**	**0.7931**	**0.7180**	**0.6530**
**LPP**	0.9270	0.6818	0.5138	0.4107	0.3435
**BDA**	0.9459	0.6652	0.4785	0.3726	0.3067
**ELPP**	0.9112	0.6120	0.4332	0.3629	0.3054
**LLE**	0.8766	0.5757	0.4211	0.3341	0.2782
**LDA**	0.8586	0.5253	0.3742	0.2937	0.2420
**ISOMAP**	0.8491	0.5064	0.3548	0.2763	0.2285
**CSVM**	0.7396	0.4269	0.2951	0.2290	0.1926

**Table 3:** Average precision of top ranked results for different methods after ninth feedback.

Methods	top10	top20	top30	top40	top50
**DFISOMAP**	**0.9660**	**0.8901**	**0.8246**	**0.7587**	**0.6965**
**LPP**	0.9543	0.7680	0.5984	0.4855	0.4073
**BDA**	0.9598	0.7534	0.5694	0.4479	0.3705
**ELPP**	0.9251	0.7021	0.5239	0.4118	0.3444
**LLE**	0.9199	0.6717	0.5078	0.4059	0.3393
**LDA**	0.9269	0.6454	0.4678	0.3689	0.3047
**ISOMAP**	0.9009	0.6093	0.4406	0.3449	0.2856
**CSVM**	0.7438	0.4649	0.3353	0.2643	0.2214

**Figure 9:**
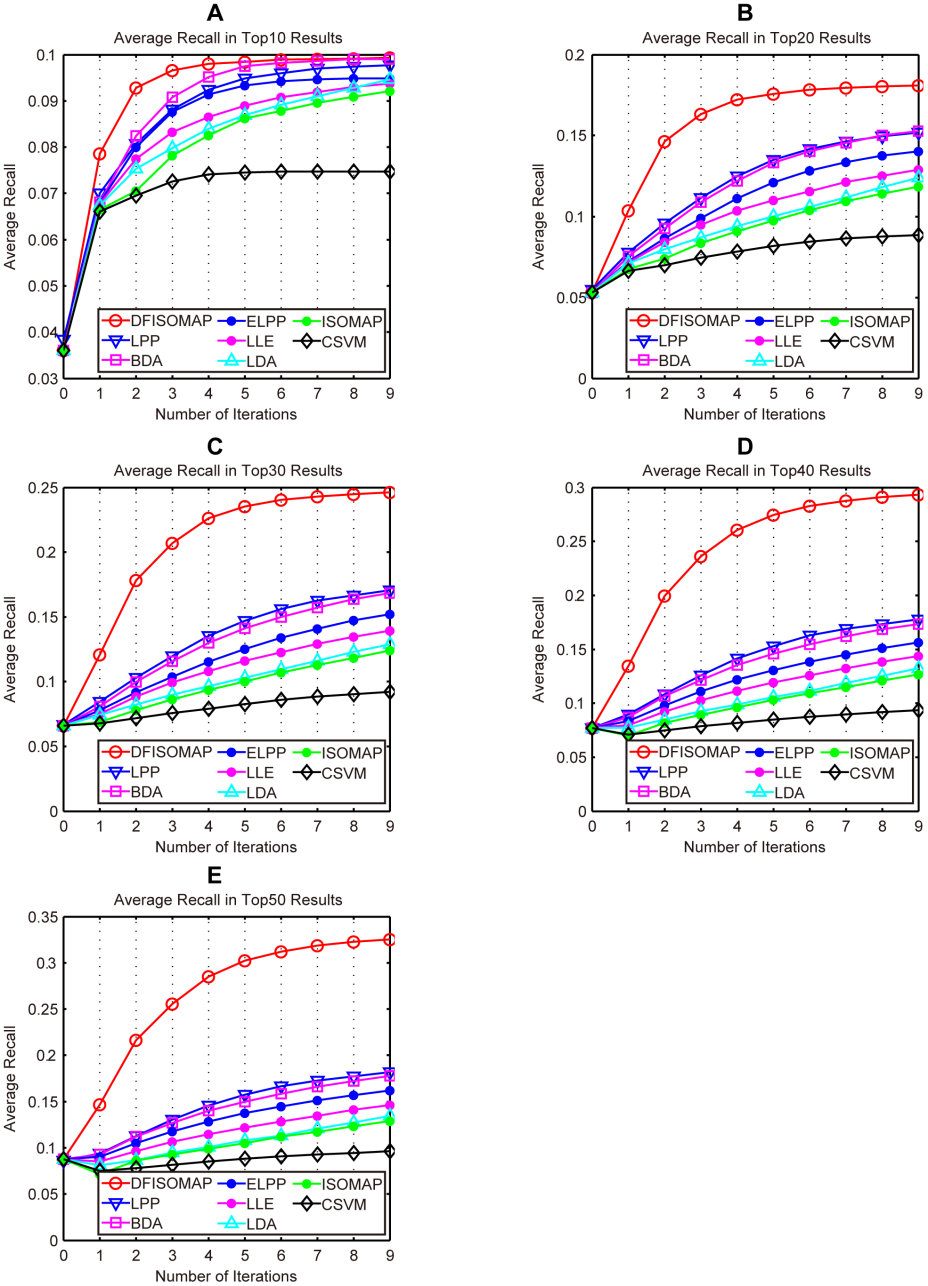
AR of DFISOMAP, LPP, BDA, ELPP, LLE, LDA, ISOMAP and CSVM. Subfigures (A), (B), (C), (D) and (E) detail AR in the top 10, 20, 30, 40, and 50 results, respectively.

**Figure 9**, **Table 4** and **Table 5** present AR of different algorithms. Specifically, **Figure 9** (A), (B), (C), (D) and (E) demonstrate AR of different approaches obtained in the top 10, 20, 30, 40, and 50 results, respectively. We can conclude from the figure that DFISOMAP is more effective than the other compared methods. Moreover, AR values of top ranked results for different methods after the fifth and ninth feedback are given in **Table 4** and **Table 5**, respectively. According to these two tables, we can see that DFISOMAP is more effective than other approaches.

**Table 4:** Average recall of top ranked results for different methods after fifth feedback.

Methods	top10	top20	top30	top40	top50
**DFISOMAP**	**0.0985**	**0.1758**	**0.2351**	**0.2746**	**0.3025**
**LPP**	0.0949	0.1349	0.1470	0.1530	0.1575
**BDA**	0.0975	0.1332	0.1411	0.1458	0.1497
**ELPP**	0.0933	0.1211	0.1252	0.1307	0.1376
**LLE**	0.0890	0.1099	0.1159	0.1193	0.1220
**LDA**	0.0869	0.1001	0.1031	0.1059	0.1083
**ISOMAP**	0.0862	0.0975	0.1001	0.1029	0.1052
**CSVM**	0.0745	0.0818	0.0828	0.0849	0.0880

**Table 5:** Average recall of top ranked results for different methods after ninth feedback.

Methods	top10	top20	top30	top40	top50
**DFISOMAP**	**0.0993**	**0.1810**	**0.2464**	**0.2931**	**0.3253**
**LPP**	0.0977	0.1519	0.1706	0.1778	0.1818
**BDA**	0.0990	0.1528	0.1682	0.1732	0.1777
**ELPP**	0.0949	0.1402	0.1522	0.1563	0.1621
**LLE**	0.0938	0.1290	0.1395	0.1437	0.1464
**LDA**	0.0947	0.1240	0.1290	0.1324	0.1348
**ISOMAP**	0.0922	0.1184	0.1242	0.1265	0.1289
**CSVM**	0.0748	0.0885	0.0920	0.0936	0.0963

### 4.5. Effects of Parameters

**(1) Effects of α.** As shown in equation (17), parameter
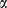
controls the contribution of 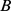 to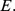 Where
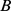
stands for utilizing LLE to preserve local geometry of positive feedback examples.

With the same experimental setup detailed above, we conduct experiments to evaluate effects of 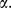
In our experiments, we increase
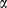
from 0 to 100 with step 10, and set
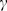
as 1400. **Table 6** and **Table 7** show AP and AR of DFISOMAP in top50 results, respectively. From which we can draw the following conclusions. 1) DFISOMAP achieves best performance when
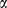
is set as 10. 2) With the increasing of 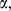
performance of DFISOMAP degrades. 3) When
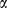
is set as 0, i.e.,
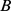
has no contribution to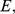 performance of DFISOMAP is worst. The conclusion verifies the effectiveness of applying LLE to minimize reconstruction error within positive feedback examples.

**Table 6:** Average precision of DFISOMAP with different
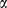
in top50 results, and
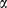
increases from 0 to 100, with step 10.

iteration	1	2	3	4	5	6	7	8	9
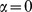	0.2866	0.4261	0.5004	0.5486	0.5796	0.6043	0.6207	0.6297	0.6369
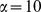	**0.3124**	**0.4770**	**0.5563**	**0.6164**	**0.6530**	**0.6720**	**0.6839**	**0.6923**	**0.6965**
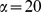	0.3105	0.4759	0.5607	0.6182	0.6488	0.6660	0.6800	0.6870	0.6952
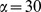	0.3086	0.4734	0.5617	0.6181	0.6477	0.6660	0.6769	0.6842	0.6886
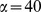	0.3072	0.4691	0.5628	0.6204	0.6527	0.6696	0.6796	0.6877	0.6931
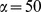	0.3063	0.4670	0.5588	0.6123	0.6442	0.6620	0.6734	0.6821	0.6869
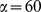	0.3056	0.4662	0.5557	0.6064	0.6374	0.6543	0.6649	0.6717	0.6766
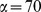	0.3047	0.4661	0.5568	0.6091	0.6386	0.6554	0.6689	0.6779	0.6835
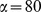	0.3042	0.4639	0.5549	0.6056	0.6358	0.6540	0.6678	0.6775	0.6827
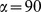	0.3033	0.4634	0.5549	0.6057	0.6352	0.6521	0.6649	0.6730	0.6782
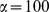	0.3026	0.4636	0.5535	0.6073	0.6359	0.6535	0.6658	0.6734	0.6789

**Table 7:** Average recall of DFISOMAP with different
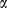
in top50 results, and
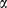
increases from 0 to 100, with step 10.

iteration	1	2	3	4	5	6	7	8	9
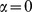	0.1336	0.1911	0.2244	0.2465	0.2608	0.2727	0.2802	0.2844	0.2879
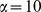	**0.1467**	**0.2167**	**0.2558**	**0.2850**	**0.3025**	**0.3123**	**0.3186**	**0.3229**	**0.3253**
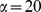	0.1459	0.2161	0.2584	0.2865	0.3018	0.3105	0.3174	0.3212	0.3258
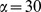	0.1452	0.2146	0.2585	0.2857	0.3005	0.3104	0.3163	0.3200	0.3222
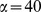	0.1447	0.2127	0.2584	0.2870	0.3025	0.3112	0.3167	0.3212	0.3242
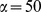	0.1443	0.2121	0.2569	0.2838	0.2992	0.3078	0.3141	0.3192	0.3217
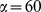	0.1440	0.2116	0.2552	0.2803	0.2952	0.3033	0.3090	0.3129	0.3155
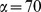	0.1437	0.2115	0.2555	0.2819	0.2965	0.3044	0.3112	0.3158	0.3188
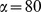	0.1434	0.2108	0.2550	0.2811	0.2962	0.3046	0.3111	0.3162	0.3191
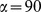	0.1431	0.2104	0.2551	0.2806	0.2954	0.3032	0.3091	0.3135	0.3163
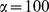	0.1428	0.2104	0.2546	0.2810	0.2954	0.3035	0.3095	0.3134	0.3162

**(2) Effects of γ.** Equation (17) demonstrates that
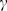
controls the contribution of 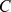 to
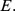
Where
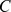
stands for similarity propagation in positive and negative examples.

With the same experimental setup mentioned above, we conduct experiments to explore effects of 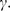
In our experiments, we increase
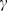
from 0 to 2000 with step 200, and set as 10. **Table 8** and **Table 9** detail AP and AR of DFISOMAP in top50 results, respectively. From the table we can draw the following conclusions. 1) DFISOMAP achieves best performance when
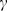
is set as 1400. 2) When
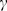
is set as 0, i.e., there is no similarity propagation, performance of DFISOMAP is worst. The conclusion confirms effectiveness of similarity propagation.

**Table 8:** Average precision of DFISOMAP with different
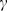
in top50 results, and
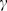
increases from 0 to 2000, with step 200.

iteration	1	2	3	4	5	6	7	8	9
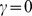	0.2166	0.2867	0.3137	0.3414	0.3562	0.3704	0.3800	0.3862	0.3895
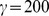	0.3053	0.4671	0.5520	0.6041	0.6354	0.6556	0.6681	0.6755	0.6800
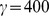	0.3080	0.4694	0.5561	0.6117	0.6444	0.6631	0.6733	0.6804	0.6853
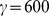	0.3096	0.4720	0.5569	0.6120	0.6470	0.6633	0.6754	0.6834	0.6879
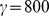	0.3111	0.4741	0.5578	0.6118	0.6466	0.6645	0.6778	0.6855	0.6920
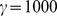	0.3114	0.4781	0.5563	0.6156	0.6508	0.6683	0.6818	0.6897	0.6953
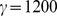	0.3118	0.4779	0.5566	0.6128	0.6470	0.6652	0.6785	0.6865	0.6923
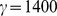	**0.3124**	**0.4770**	**0.5563**	**0.6164**	**0.6530**	**0.6720**	**0.6839**	**0.6923**	**0.6965**
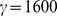	0.3130	0.4779	0.5548	0.6123	0.6482	0.6665	0.6778	0.6859	0.6914
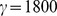	0.3132	0.4775	0.5538	0.6120	0.6476	0.6672	0.6790	0.6876	0.6932
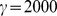	0.3137	0.4776	0.5546	0.6083	0.6428	0.6618	0.6749	0.6810	0.6859

**Table 9:** Average recall of DFISOMAP with different
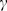
in top50 results, and
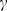
increases from 0 to 2000, with step 200.

iteration	1	2	3	4	5	6	7	8	9
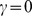	0.1060	0.1337	0.1448	0.1557	0.1618	0.1673	0.1705	0.1728	0.1741
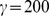	0.1439	0.2122	0.2530	0.2787	0.2941	0.3036	0.3098	0.3139	0.3166
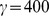	0.1449	0.2136	0.2549	0.2822	0.2979	0.3075	0.3130	0.3171	0.3199
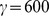	0.1456	0.2144	0.2563	0.2836	0.3008	0.3091	0.3153	0.3196	0.3220
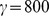	0.1461	0.2153	0.2565	0.2831	0.2994	0.3085	0.3157	0.3201	0.3237
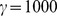	0.1463	0.2168	0.2555	0.2848	0.3015	0.3102	0.3173	0.3217	0.3248
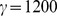	0.1464	0.2169	0.2560	0.2835	0.3005	0.3098	0.3169	0.3209	0.3242
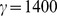	**0.1467**	**0.2167**	**0.2558**	**0.2850**	**0.3025**	**0.3123**	**0.3186**	**0.3229**	**0.3253**
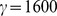	0.1469	0.2172	0.2556	0.2837	0.3011	0.3100	0.3156	0.3196	0.3227
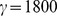	0.1470	0.2170	0.2551	0.2834	0.3008	0.3106	0.3167	0.3214	0.3247
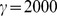	0.1472	0.2171	0.2554	0.2818	0.2988	0.3084	0.3152	0.3186	0.3213

## Conclusion

Starting from the assumption that medical images are artificially embedded in a high-dimensional visual feature space, we propose the dual-force ISOMAP (DFISOMAP) to map medical images from high-dimensional feature space to low-dimensional embedding. In the framework of CBMIR, DFISOMAP precisely preserves the geometric structure of positive feedback examples according to the ISOMAP criterion, and effectively separates negative examples from positive examples by utilizing two forces. The evaluation results on a subset of the IRMA medical image dataset show that DFISOMAP outperforms popular dimensionality reduction-based RF algorithms, e.g., LDA, BDA, LPP, ISOMAP, LLE, ELPP and support vector machine-based RF algorithms, e.g., CSVM.

## Supporting Information

Appendix S1Proof of 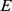 is symmetric.(DOC)
