# Supplementary material for: Dual-Force ISOMAP: A New Relevance Feedback Method for Medical Image Retrieval
Source: PLoS One. 2013 Dec 31;8(12):e84096. doi: 10.1371/journal.pone.0084096 (PMC3877227; doi:10.1371/journal.pone.0084096)
Supplement: Appendix S1 — Proof of is symmetric. (DOC) [file pone.0084096.s001.doc]

**Appendix S1:** Proof of $E$is symmetric

1. Proof of $A$ is symmetric

$$\begin{matrix} A^{T}=(X_{+}\tau(D_{G})X_{+}^{T})^{T} \\ =X_{+}\tau(D_{G})^{T}X_{+}^{T}. \end{matrix}$$

Because

$$\begin{matrix} (\tau(D_{G}))^{T}=-\frac{1}{2}(H\tilde{D}_{G}H)^{T} \\ =-\frac{1}{2}H^{T}\tilde{D}_{G}^{T}H^{T} \\ =-\frac{1}{2}H\tilde{D}_{G}^{T}H \\ =-\frac{1}{2}H\tilde{D}_{G}H \\ =\tau(D_{G})\text{,} \end{matrix}$$

so $A=A^{T}\text{,}$ $A$ is symmetric.

1. Proof of $B$ is symmetric

$$\begin{matrix} B^{T}=(X_{+}(I-W^{T})(I-W^{T})^{T}X_{+}^{T})^{T} \\ =X_{+}(I-W^{T})(I-W^{T})^{T}X_{+}^{T} \\ =B\text{,} \end{matrix}$$

so $B$ is symmetric.

1. Proof of $C$ is symmetric

$$\begin{matrix} C=(X(L-M)X^{T})^{T} \\ =X(L-M)^{T}X^{T} \\ =X(L^{T}-M^{T})X^{T} \\ =X(L-M)X^{T} \\ =C\text{,} \end{matrix}$$

so $C$ is symmetric.

Then

$$\begin{matrix} E^{T}=(A-\alpha B+\gamma C)^{T} \\ =A^{T}-\alpha B^{T}+\gamma C^{T} \\ =A-\alpha B+\gamma C \\ =E. \end{matrix}$$

Therefore$E$is symmetric.
